# The interplay between oxidative stress and autophagy: focus on the development of neurological diseases

**DOI:** 10.1186/s12993-022-00187-3

**Published:** 2022-01-29

**Authors:** Marjan Talebi, Seyyed Ali Mohammadi Vadoud, Alireza Haratian, Mohsen Talebi, Tahereh Farkhondeh, Ali Mohammad Pourbagher-Shahri, Saeed Samarghandian

**Affiliations:** 1 Department of Pharmacognosy, School of Pharmacy, Shahid Beheshti University of Medical Sciences, Tehran, Iran; 2 Department of Pharmaceutical Biotechnology, School of Pharmacy, Shahid Beheshti University of Medical Sciences, Tehran, Iran; 3 Department of Chemistry and Biochemistry, University of Texas at Arlington, Arlington, TX 76019 USA; 4 Viatris Pharmaceuticals Inc, 3300 Research Plaza, San Antonio, TX 78235 USA; 5 Medical Toxicology and Drug Abuse Research Center (MTDRC), Birjand University of Medical Sciences, Birjand, Iran; 6 Faculty of Pharmacy, Birjand University of Medical Sciences, Birjand, Iran; 7 Noncommunicable Diseases Research Center, Neyshabur University of Medical Sciences, Neyshabur, Iran

**Keywords:** Autophagy, Oxidative stress, Neurological diseases, Neurodegenerative diseases, Signaling pathways, Alzheimer’s disease, Parkinson’s disease, Reactive Oxygen Species (ROS)

## Abstract

Regarding the epidemiological studies, neurological dysfunctions caused by cerebral ischemia or neurodegenerative diseases (NDDs) have been considered a pointed matter. Mount-up shreds of evidence support that both autophagy and reactive oxygen species (ROS) are involved in the commencement and progression of neurological diseases. Remarkably, oxidative stress prompted by an increase of ROS threatens cerebral integrity and improves the severity of other pathogenic agents such as mitochondrial damage in neuronal disturbances. Autophagy is anticipated as a cellular defending mode to combat cytotoxic substances and damage. The recent document proposes that the interrelation of autophagy and ROS creates a crucial function in controlling neuronal homeostasis. This review aims to overview the cross-talk among autophagy and oxidative stress and its molecular mechanisms in various neurological diseases to prepare new perceptions into a new treatment for neurological disorders. Furthermore, natural/synthetic agents entailed in modulation/regulation of this ambitious cross-talk are described.

## Introduction

Neurological diseases contribute to 2% of the global burden of diseases. Activation and inhibition of autophagy play a central role in neurological disorders [1]. Various types of diseases accompanying neuronal degeneration, cerebral ischemia, and neurotoxicity are examples of communal neuronal disorders with approximately high prevalence [2].

Autophagy is a Greek term consisting of “auto’’ that means “self” and “phage” that means “to eat” that refers to an evolutionary-conserved process [3]. Autophagy which is regarded as a catabolic pathway is a degradation process of intracellular organelles and proteins by lysosomes. Overall the mechanism of autophagy evolves cleansing property by removing all the degraded products and retaining the valuable component for the survival of the body [4–7]. Different regulators support and coordinate the autophagy process like the transcription factor gene etc. It also involves the homeostatic process if a change occurs due to functional genetic and over-excretion. There is a considerable accumulation of pathogenic protein in NDD, so dysfunctional autophagy occurs in this type of disease. There is a mutation in the autophagy regulation gene that induces NDD [8].

Although Autophagy is an essential mechanism used for the digestion of damaged organelles and molecules, abnormalities in this process have proved to be the onset of some critical diseases such as cancer and NDDs [9].

It is noteworthy to state that there are three diverse types of autophagy with reference to their modes of delivery known as chaperone-mediated autophagy (CMA, autophagy mediated by particular cytosolic proteins that encompass a pentapeptide motif), microautophagy (autophagy mediated by lysosomal action), and macroautophagy (autophagy in cells form double-membrane vesicles, known as autophagosomes, nearby a portion of cytoplasm) [9]. Notably, the main difference between macroautophagy, chaperone-mediated autophagy, and microautophagy is that in chaperone-mediated autophagy and microautophagy, direct cargo delivery to lysosomes is observed but in macroautophagy, the double-layered membrane vesicles are transferred to lysosomes. Macroautophagy engulfs accumulated proteins, long-lived proteins, pathogens, and degraded organelles with the cytoplasm. They are covered with two membranes named autophagosomes to intercalate with the lysosomes. This is degraded to recycle large molecules for reuse. Microautophagy is a process in which lysosomes engulf very few amounts of the cytosolic substrate. Chaperone-mediated autophagy is triggered from stress like starvation with heat shock cognate protein having a pentapeptide sequence containing KFERQ [10–12].

The autophagic degradation process is commenced when some cytosolic components sequester and form a cup-shaped structure termed isolation membranes. Next, the isolation membranes (also called phagophores) are elongated and finally sealed and turned into double-layered membrane vesicles called autophagosomes transferred to the lysosome for degrading steps [13].

Reactive oxygen species (ROS) have been involved in the pathogenesis of NDDs. High concentrations of ROS cause oxidative damage in the brain of patients with neurodegenerative conditions [14]. Different types of stresses affect ROS levels and, therefore, autophagy, including cellular stress, ischemia–reperfusion, hypoxia, nutritional deprivation, and exogenous substances [15].

To the best of our knowledge, neurological disease is mainly leading to incapacitated individuals. Moreover, due to the multi-faced pathology of these diseases discovery, an essential mechanism like the interplay between ROS and autophagy will be targeted in prospective therapeutic approaches [16].

We aimed to discuss autophagy and its molecular regulation, its relation with oxidative stress in neurological disorders, and a brief overview of various therapeutic implications of autophagy in the current review article. Moreover, various natural/synthetic putative therapeutic medications with the amazing potential of regulating the autophagy/ROS axis are discussed.

## Molecular regulation of autophagy

The molecular mechanisms attributed to the occurrence of autophagy are widely researched. The genes playing significant roles in this process are denoted as *ATG*s (autophagy-related). At least there are 30 ATG genes have been identified in yeasts which there are many mammalian monologues for them. Among the *ATGs* genes, *ATG 1–10*, *12–14*, *16–18*, *29*, and *31* (called *AP-ATG*) are pivotal for autophagosome generation. *AP-ATG* proteins can be divided into six types, originate on their functionality: the ATG1 protein kinase and its regulators, *ATG2-ATG18* complex, the *ATG8* complex, the *ATG12* complex, *ATG 9*, and phosphatidylinositol-3-kinase (*AS-PI3K*) complex. In normal homeostasis (when starvation is not applied), one of the main inhibitors of autophagy induction is the target of rapamycin (TOR) [17]. In the autophagosome formation step, the primary regulators are class III phosphatidylinositol-3-kinase (*AS-PI3K*) complex and *ATG6*. Two ubiquitin-like conjugation systems are in charge of autophagosomal membrane elongation: the *ATG5* and *ATG12* complex, which are localized on phagophore with *ATG16*, and the *ATG8*-phosphoethanolamine (PE) complex, which could be found on both phagophore and autophagosomal membrane [18, 19]. The *ATG8-PE* complex can be deconjugated by *ATG4* protease in a ROS-regulated step, enabling the recycling of this protein. *ATGs*, AMP-activated protein kinase (AMPK) pathways, and mammalian target of rapamycin (mTOR) (have an incredible character in autophagy regulation [20].

The mTOR acts as a main effector in cerebrovascular dysfunction in AD. The mTOR is involved in brain vascular and cerebral blood flow impairment, leading to cognitive dysfunction. Additionally, mTOR induces damage in the blood–brain barrier (BBB), leading to disruption in BBB integrity and cognitive defect [20].

Unc-51-like autophagy activating kinase 1 (*ULK1*) is *ATG1* homologous which promotes autophagy [21]. Besides, Beclin-1 is an essential protein that acted in the autophagy progression. Beclin-1 affects the autophagy process via management of vacuolar protein sorting 34 (Vps34) and generation of a complex comprising Beclin-1–Vps34–Vps15. Remarkably, Beclin-1 is not acted only in the autophagy commencement, but it also provides to maturation and biogenesis of autophagosome. Starvation is one of the well-distinguishing elicitors of autophagy, in which mTOR Complex 1 (mTORC1) is prevented. As a final point, this complex prompts phagophore creation [22–24]. Mitophagy is the best type of autophagy and is chiefly managed by microtubule-related protein 1 light chain 3 (*MAP1LC3*; mostly defined as LC3)-related autophagy receptors through Ub-independent and Ub-dependent pathways [25].

## The relationship between reactive oxygen species and autophagy

ROS are a large group of highly reactive-oxygen-containing species that comprise free radicals, oxygen anions, and hydrogen peroxide and are known for their short lives and high reaction desire [26, 27]. Hydrogen peroxide is now believed to be required for insulin, cytokines, growth factors, and some signaling pathways; therefore, their regulating characteristics in biological processes are almost undeniable [28, 29]. The metabolism of oxygen is supposed to be the main pathway for ROS production. In addition, ROS at low levels is involved in cellular signaling pathways. However, on the other hand, they can cause severe irreversible damages to proteins, DNA, and lipids at high levels of ROS existence [30]. Interestingly, a growing level of evidence proposes which ROS are witnessed as crucial signals for activation of autophagy following numerous stimuli. The high and moderate ROS amounts can precisely begin mitophagy. However, only a high amount of ROS can induce common autophagy. There is almost enough evidence that supports the relationship between excessive ROS levels being generated due to stressful conditions like starvation or hypoxia and the autophagy process [31].

Herein, the molecular signaling cascades that contributed to the accomplishment and initiation of autophagy subsequent disclosure to ROS are sophisticated. These mechanisms are mainly entailed nuclear transcriptional progress and cytoplasmic posttranscriptional progress. These transcriptional mechanisms frequently comprise the activation of p53, HIF-1, and Nrf2, then autophagy modulation occurs [32, 33]. Several studies have suggested ROS-mediated autophagy via mTOR pathways in the cytoplasm. The mTOR activation, which occurs in the presence of enough nutrients and amino acids, results in phosphorylation of *ATG13*, which in turn prevents the autophagic process. As a result of starvation, inhibition of mTOR leads to activation of phosphatases that increase the number of dephosphorylated *ATG13* and the formation of autophagosomes [34, 35]. During nutrient deficiency, the demand for more ATP production from mitochondria is increased; therefore, more ROS are produced. It was demonstrated that hydrogen peroxide as a product of starvation-induced autophagy interacts with an essential cysteine residue located on the catalytic site of HsATG4. This cysteine is a critical point in autophagy regulation by attaching *ATG8* to the autophagosomal membrane, a hallmark of the autophagic process. H_2_O_2_ and O_2_^−^ produced by mitochondria are mostly available ROS to provoke autophagy. The existence of elevated levels of H_2_O_2_ inactivated autophagy through the PI3K/Akt signaling pathways can be reactivated by hindering PTEN and Akt or mTORC1. In the same way, excessive H_2_O_2_ can persuade autophagy via an AMPK-dependent gate and is along with the deterioration of mTORC1 activity. Phosphorylation of *ULK1/ATG1* conjugate by AMPK is another crucial role of this protein in the induction of autophagy in starvation conditions. Several stress response proteins, for instance, c-Jun N-terminal kinase (JNK), extracellular regulated kinase (ERK), and p38MAPK, are also concerned with inducing autophagy in the face of the high levels of ROS [13]. Notably, *ATG13* phosphorylation is a primary factor in the autophagy pathways. Two serine sites in *ATG13* have been identified, which in starvation condition are phosphorylated by mTOR and AMPK pathway, resulting in an autophagic response. AMPK is also a regulator for *TSC2*, a tumor suppressor gene involved in repressing the kinase mTOR in the mTORC1 complex. In elevated levels of ROS, *TSC2* is activated by a cellular damage sensor ATM (Ataxia telangiectasia mutated) through an AMPK mediated pathway which ends in mTORC1 repression and autophagy induction [36]. In a manner of accumulating these foundations, the interplay between ROS and autophagy is a double-edged sword [37].

Besides, another approach is that autophagy has been recommended as a possible survival mechanism against ROS formation by eradicating damaged or laid-off agents to avoid unnecessary oxidative injury [38]. The Keap1-Nrf2 pathway is shown as a protection pathway upon encountering oxidative stress [39, 40]. The p62 for short or p62/SQSTM1 protein may involve information of autophagosome as a receptor or an autophagic adaptor. Phosphorylation of p62 in the mTORC1 autophagy signaling pathway elevates the ubiquitinated cargos and phosphorylated Keap1 integration, which is vital for degradation of the Nrf2 [41].

**Fig. 1:**
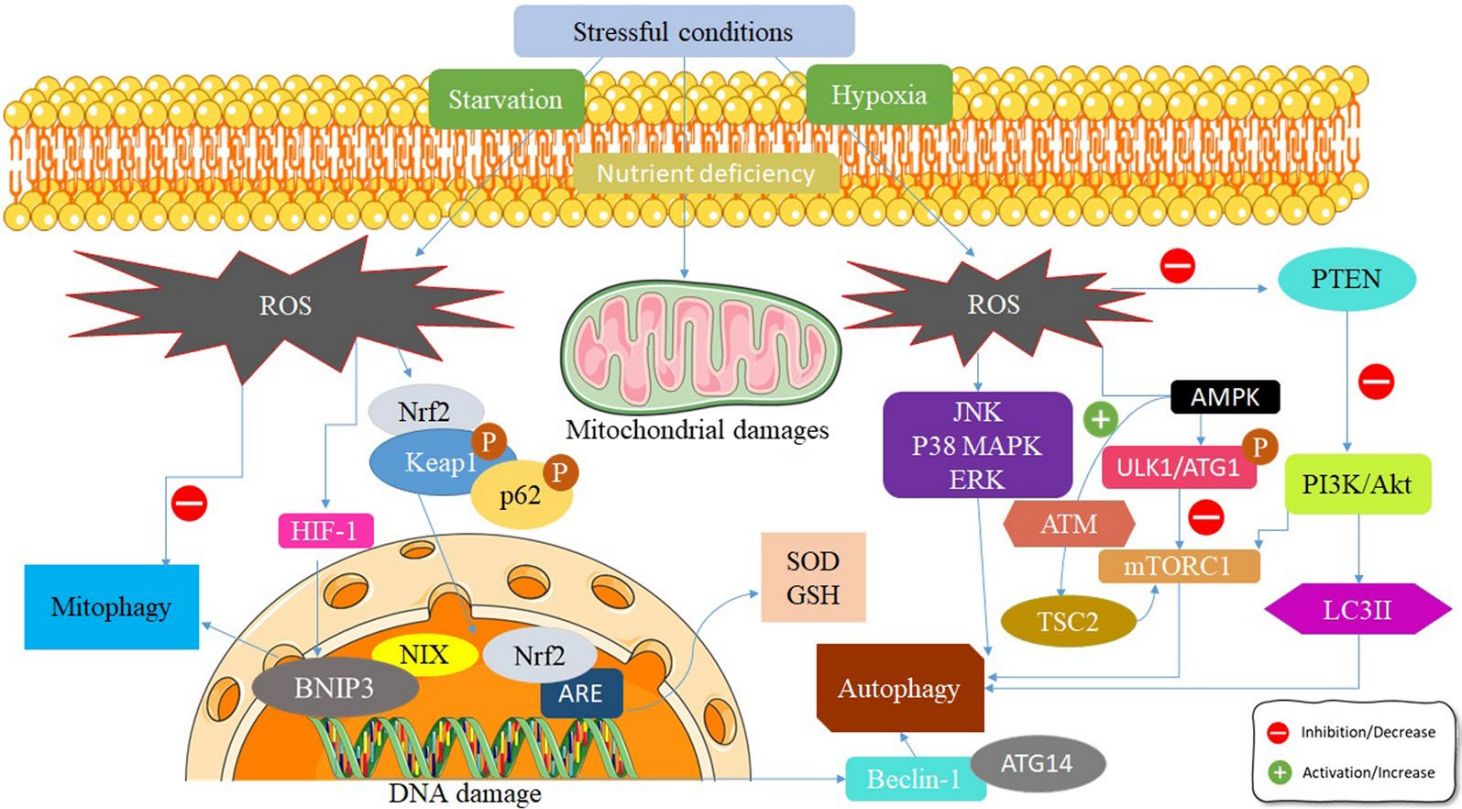
The interplay between ROS and autophagy. ROS can be induced following various triggers entailing hypoxia, starvation, and mitophagy. Afterward excessive production of ROS, some factors are affected comprising HIF-1α, AMPK, JNK, and Nrf2/Keap1. Alongside these biological circumstances, autophagy may be inhibited/activated by forming the ULK complex, PI3K complex, and mTORC1 complex. Important factors involved in autophagy are LC3II and p62

In summary, in a well-established review article conducted by Zhao and coworkers, six involved pathways in the diverse cell fates by the contribution of ROS generation in mitochondria. These cascades are stated as a) ROS-ATG4-LC3-II, b) ROS-TIGAR, c)ROS-Nrf2-p62, d) ROS-HIF-1-BNIP3/NIX, e) ROS-FOXO3-LC3/BNIP3, and f) ROS-AMPK-ULK1 complex [42] (Fig. 1.).

## Autophagy and ROS in neurological diseases

There are many relations observed between neurodegenerative disorders and autophagy. Autophagy is dynamic for cerebral homeostasis, and whenever its regulation disturbances happen, it causes neurodegenerative effects. Generally, autophagy is vital for the differentiation, growth, maintenance, and homeostasis of neuronal cells. The aggregation of toxic and unwanted degraded molecules with pathological is vital in NDDs, comprising Parkinson’s (PD) and Alzheimer’s (AD) diseases. The neurodegenerative disorder is due to the activation of autophagy genes or autophagosomal degradation in response to stress/injury in cerebral tissues [43]. There is evidence of post-mortem brain image through electron microscopy. The irregularities in the endosomal lysosomal pathways and autophagosomal accumulation happen due to the neuronal loss by autophagy in the mouse model [44].

There are two approaches for autophagy acting via mTOR independent and dependent mechanisms. The modulation of mTOR dependent approach is through rapamycin inhibiting mTORC1 for various NDDs. The primary function of rapamycin is immune-suppressant and second as anti-proliferative property, which is causing benefit for the chronic neurodegenerative disorder. It is also observed a good response in the preclinical model. Further mTOR independent modulators for autophagy are anticipated for therapeutic efficacy by activation of AMPK molecules. Cells are not directly involved in the autophagy mechanism. They are associated with different receptors like cAMP and alpha receptors [8].

Oxidative damage is found in NDDs leading to increased autophagic activity [45]. During these conditions, autophagy cannot act as a cell-survival mechanism but stimulate cell death. In AD conditions, oxidative stress induces neuronal cell death via stimulating autophagy of accumulated Aβ and permeabilization of the lysosomal membrane leading to neuron death. Oxidative stress-induced-mitochondrial damage in pyramidal neurons is considered as autophagic degradation in AD (mitophagy), resulting in neurodegeneration. Improper expression of protein phosphatase 2A (PP2A) is related to the onset of some NDDs via an increase in susceptibility to oxidative stress-mediated cell death [46].

Oxidative stress also can stimulate autophagy in neurons via downregulating Oxi-α (a neuroprotective protein in dopamine neurons). In a normal cell, Oxi-α induces mTOR and subsequently decreased autophagy. However, overproduction of ROS decreases the expression of Oxi-α and resulting in a decrease in mTOR activity, leading to autophagy [47].

A summary of compounds affecting autophagy in neurological diseases is stated in Table 1.

**Table 1:** Summary of compounds involved in regulation (activation or inactivation) of autophagy process of neurological diseases

Neurological disease	Therapeutic compound	Species/cell lines	Primary contributed mechanisms related to autophagy	Refs
Alzheimer’s disease	Lithium	Various models	Regulation of inositol monophosphatase, GSK-3β, and mTOR	[161]
	Hydroxyurea	APP/PS1 mice	Augmentation of the expression of LC3	[162]
	Atorvastatin	Aβ1–42- SH-SY5Y	Elevation of the expression of sestrins and LC3-II, alleviation of sirtuins and TPP1	[163]
	Ferric-Tannic nanoparticles	In vitro	activation of lysosome	[164]
	Curcumin	APP/PS1 double transgenic AD mice	Inhibition of PI3K/Akt/mTOR, an increase of LC3I/II and Beclin1 expression	[165]
	Berberine	Three × Tg-AD mice	Augmentation of LC3-II, Beclin-1, hVps34, and Cathepsin-D	[166]
	Berberine and Curcumin	Aβ1-42-AD mice	Augmentation of AMPKα phosphorylation and cell autophagy	[65]
	Resveratrol	Aβ1–42-induced AD-PC12	Activation of mitophagy due to the abrogation of oxidative stress	[167]
	Geniposide	APP/PS1 mice	downregulation of mTOR	[168]
	Polydatin	Aβ-induced neuron cytotoxicity	activation of AMPK/mTOR pathway	[169]
	Melatonin	AAV-hTau^P301L^ viral vectors and okadaic acid-Tauopathy, mice and human brain tissue	improvement of the autophagy markers comprising p62, LAMP1, and LC3	[170]
	α-Tocopherol	Aβ-SH-SY5Y	Regulation of cathepsin-B, -L, -D, syntaxin 17, GABA type A receptor-associated protein, GABA type A receptor-associated protein-like 1; ATG3, ATG4A, ATG4B, myotubularin related protein 3, UV radiation resistance-associated, autophagy and Beclin 1 regulator 1, vacuole membrane protein 1, WDrepeat domain, phosphoinositide interacting 1, WDrepeat domain, phosphoinositide interacting 2, unc-51 like autophagy activating kinase 2	[171]
	Oleuropein aglycone	Aβ-AD mice, SH-SY5Y	Induction of AMPK/ULK1, inhibition of mTOR	[172, 173]
	Garcinol	Acrylamide-brain of zebrafish larvae	Regulation of cathepsin-B	[174]
	Carnosic acid	Aβ 1–42- SH-SY5Y	Increase LC3-II/I ratio, decrease SQSTM1(p62)	[175]
	β-asarone	Aβ, PC12 cells	Alleviation of autophagy occurred via Akt/mTOR signaling pathway	[176]
	Euxanthone	Aβ1-42-PC12	Augmentation of LC3-II and Beclin1, alleviation of p62	[177]
	Oleocanthal	TgSwDI mice	Activation of AMPK/ ULK1 pathway	[178]
	*Ganoderma lucidum*	MPTP-induced PD in mice	Elevation of NIX expression, decrease of LC3-II/LC3-I ratio, regulation of AMPK, mTOR, and ULK1	[179]
Parkinson’s disease	Metformin	In vitro and in vivo	Activation of AMPK and inhibition of α-synuclein	[180]
	Sulforaphane	Rotenone-mice, SH-SY5Y	Decrease expression of LC3-II	[181]
	Morphine (low dose)	6-OHDA-SH-SY5Y and rats	Augmentation of LC3-II, alleviation of p62	[182]
	Manganese nanoparticle	N27 dopaminergic neuronal cells	Improvement of Beclin 1 and LC3	[183]
	α-Arbutin	Rotenone-induced PD, SH-SY5Y, drosophila	Modulation of AMPK/p62	[184]
	Polydatin	Rotenone or Parkin deficiency-SH-SY5Y	Promotion of ATG5 in a parkin-independent autophagy manner	[185]
	Glycyrrhizic acid	6-OHDA and corticosterone-induced PD in SH-SY5Y	Attenuation of α-Syn and p-S1292-LRRK2 proteins expression, upregulation of LC3B II/I and Beclin-1	[186]
	α-lipoic acid	6-OHDA-SH-SY5Y cells	blockade AMPK/mTOR signaling pathway	[187]
	Icariin	Rotenone-PC12 cells	Regulation of LC3-II, Beclin1, p62	[188]
	β-amyrin	6-OHDA- *Caenorhabditis elegans*	Regulation of LGG-1	[189]
	kaempferol	Rotenone- SH-SY5Y	Increase the expression of LC3-II	[190]
Huntington’s disease	Tolfenamic acid	transgenic R6/1 mice	Increase LC3-II/LC3-I ratio, decrease expression of p62	[191]
	Liraglutide	mHTT- SK-N-MC cells	Upregulation of the phosphorylation of Thr172-AMPK and LC3-II	[192]
	Trehalose	skin biopsies of HD patients	Increase LC3 and LAMP2-A levels	[193]
	Resveratrol	polyQ-Htt- SH-SY5Y	restoring ATG4 level, allowing the LC3 lipidation, facilitating polyQ-Htt degradation	[194]
	Rutin	*Caenorhabditis elegans*	Autophagy by activation of protein degradation	[195]
Amyotrophic lateral sclerosis	Riluzole	HeLa cells	Increase the amount of HSF1 regarding the chaperone-mediated autophagy	[196]
	p-Coumaric Acid	SOD1mut- N2a cells	increased the level of LC3-II, decrease the protein level of p62	[197]
Cerebral ischemia	Ulinastatin	A variety of models	Inhibition of neuronal autophagy	[198]
	Tanshinone IIA	OGD/R- HT-22 cells	Activation of PI3K/Akt/mTOR pathway	[112]
	Resveratrol	cerebral ischemia rats	Increase expression of LC3II	[199]
	Gabapentin	middle cerebral artery occlusion-rats	Regulation of the PI3K/Akt/mTOR signaling pathway	[200]
	Melatonin	I/R-rats, OGD/R-PC12 cells	Reduction of LC3II/LC3I, an increase of p62	[201]
	*Lycium barbarum* polysaccharide	OGD/R- primary hippocampal neurons	Activation of PI3K/Akt/mTOR pathway	[202]
	Tetrahydroxystilbene glucoside	middle cerebral artery occlusion-mice	Elevation of Beclin 1 and the LC3BII/I ratio	[203]
	Shengmai	Cerebral I/R injury-mice	modulation of the AMPK, mTOR, and JNK pathways, inhibition of Beclin1 and LC3	[204]
	Esculetin	transient bilateral typical carotid artery occlusion -treated mice	Regulation of Bnip3, Beclin1, Pink1, parkin, and the LC-3 II/I ratio	[205]
	Luteolin	MCAO rat model	Regulation of SIRT3/AMPK/mTOR Signaling Pathway	[106]
Chronic Cerebral Hypoperfusion	Resveratrol	CCH-rats	Regulation of AKT/mTOR Signaling	[206]
Spinal Cord Injury	Omega-3 fatty acids	Rodent models	Increase of LC3-II expression, reduction of p38 MAPK expression	[207]
	Calcitriol	laminectomy and spinal cord compression injury-rats	Augmentation of LC3-II and Beclin1, alleviation of p62	[208]
Spinocerebellar ataxia	Caffeic acid and resveratrol	mutant ataxin-3-SK-N-SH-MJD78 cells, Drosophila	Upregulation of p62 expression	[209]
	lactulose and melibiose	SCA3 ATXN3/Q75-GFP cell model	Regulation of autophagy	[210]
Neurotoxicity	Methylone and MDPV	β-keto amphetamines-SH-SY5Y cells	Increase expression of LC3-II	[154]

### Alzheimer’s disease

Alzheimer’s disease is commonly well-identified as one of the most prevalent diseases contributed to neurodegeneration, hallmarked by a progressive loss of memory and cognition deficits. AD is characteristically described by the existence of extracellular amyloid *β* (A*β*) plaques and intracellular tau (*τ*) protein tangles as the principal biomarkers. A*β* is produced by the assistance of the gate attributed to the enzymatic cleavage of the amyloid precursor protein (*APP*) [48, 49]. Oxidative stress is a conspicuous etiological factor responsible for AD pathogenesis, associated with the establishment of the A*β* plague, phosphorylation of *τ* protein, and subsequently the development of the neurofibrillary tangles (NFTs) [27, 50]. Dysregulation of autophagy is responsible for the accumulation of pathogenic proteins that contributed to neurodegeneration such as alterations in ubiquitinated proteins, degradation of A*β*, and phosphorylation of *τ* protein. The gathering of A*β* leads to perceiving of diminished fusion of autophagosomes with lysosomes. Autophagy is taking part in the secretion of A*β* into the extracellular surroundings, wherever it fabricates the plaques. The omission of *ATG7* in *APP* transgenic mice causes downregulation in secretion and formation of A*β*. A mutation in Presenilin1 (*PSEN1*), which is entailed in cleavage of *APP*, elucidates one of the important features related to AD and leads to the deficiency of lysosome functional mission and the A*β* accumulation. Furthermore, *PSEN1* plays a part as an ER chaperone for the V0a1 subunit of lysosomal v-ATPase. The witnessed mutation damages lysosomal v-ATPase maturation and consequently surges lysosomal pH. Hence, the accumulation of *τ* protein into intracellular tangles is likewise a hallmark that contributed to AD pathogenesis [51]. Hyperphosphorylated *τ* protein co-localizes with p62 and LC3B-II in AD patients and more neuronal disturbing conditions for instance corticobasal degeneration and progressive supranuclear palsy. Additionally, abnormal *τ* proteins induce a disturbance in axonal vesicle transport via the impeding of complex, so resulting in the elevation of amount of autophagosomes in AD [52]. As Hsc70 interrelates with *τ* in the monomeric, oligomeric, and aggregated arrangement and clears by the CMA pathway, autophagy can be selective. Lysosome-associated membrane proteins-2A (LAMP-2A) is the crucial agent involved in CMA and interacts with the Hsc70- *τ* complex and activates the downstream cascade. Besides, chronic stress or environmental risk factors and augmented glucocorticoid levels have the capability to simulate AD and other coherent dysfunctions. Augmentation in levels of glucocorticoid may possibly enable the A*β* plague formation and *τ* protein phosphorylation which can be mediated by mTOR-reliant impeding of autophagy [53–56]. Thus, it is also linked to glucocorticoid-persuaded oxidative stress. By considering recent research works, it is now comprehended that autophagy change and stress-related constituents can feasibly be managed by numerous RNA binding proteins (RBPs) such as DEAD-box 5 (DDX5), fused in sarcoma (FUS), Ras GTPase-activating protein-binding protein 1 (G3BP1), poly (A)-binding protein (PABP), and T-cell intracellular antigen 1 (TIA-1). The level of the aforementioned proteins is raised in response to chronic stress and encountering chronic amounts of glucocorticoid. Above and beyond, these RBPs are appeared to be interconnected to oxidative stress reactions. For that reason, they could be considered as insightful therapeutic targets for averting the development of stress granules in AD and other *τ* pathologies. Regarding the study mentioned above conducted by Huang and colleagues, circular RNAs (circRNAS) can regulate pathological progressions by binding to RBPs, sponging miRNAs, modulating mRNA, aiding as biomarkers, and potential therapeutic targets AD [57]. Lower expression of Beclin-1 was found in the brain of an AD patient also affects autophagy. Emerging evidence proposes that spatial learning and memory deficits in AD may be strongly interrelated with the deficiency of the Nrf2-ARE pathway. Amazingly, some researchers have recommended that the interaction between oxidative stress and mitochondrial dysfunction is feasibly convoluted in AD progression owing to the impact of oxidative stress on mitochondrial transport [54]. The autophagic elimination of impaired mitochondria and A*β* mediated by Parkin can diminish oxidative stress and reinstate the energy source to delay or avoid neurodegeneration in AD transgenic mice [58]. Du and coworkers presented a study focused on gene therapy-mediated PINK1 overexpression, which could enhance the clearance of impaired mitochondria via increasing activation of autophagy receptors containing OPTN and NDP52, which in this manner led to alleviation of A*β*-induced synaptic loss and cognitive deficit in AD mice [59]. Li and colleagues hypothesized that mTOR might mediate glucagon-like peptide-1 therapy in AD [60].

It has been observed that *ATG12* and *ATG5*, Beclin1, and LC3-II increased considerably, whereas p62 expression reduced in the *τ* hyperphosphorylation rodent model of AD [61].

Activation of AMPK is provocative in the deposition of A*β* and phosphorylation of τ; however, it can ameliorate autophagy, restore mitochondrial health, mitigating insulin resistance, and abrogate oxidative damage [62].

Zhang and colleagues found that overexpression of transcriptional factor EB (TFEB) attenuated AD progression by decreasing the accumulation of A*β *via regulation of the autophagy-lysosome pathway entailing LAMP-1, cathepsin D, LC3-II, and p62 and alleviating Aβ-induced ROS generation and cell fate [63]. Diminishing the p62 protein level can disturb Nrf2, cyclic AMP, and NF-κB signaling pathways, increasing oxidative stress during AD [21]. Omata and coworkers discussed that expression of some autophagy-related genes such as *ATG1*, *ATG8A*, and *ATG18* is regulated by foxo/sir2-mediated aging procedures in AD [64].

Trichostatin A (histone deacetylase inhibitor) improved the expression of *Nrf2* and its downstream genes, thus augmenting the total antioxidant capacity of SH-SY5Y cells and hindering A*β* peptide-induced autophagy [65].

AD condition is accompanied by increase in the expression of beclin 1, ATG5, ATG12, LC3-II and p62.The effect of autophagy regulation by captopril and its effect on reducing a neurotoxic effect of prion peptide (PrP) that exerts its neurotoxic effects through changes in calcium metabolism has been explored. Captopril reduced PrP Fragment 106–126 neurotoxicity cell death by activating AMPK and blockage of autophagy, which elevated the proteins LC3-II and p62. Stimulation of LC3-II and p62 expressions could inhibit the expression of NFKB, Nrf2 and cAMP and increase ROS production resulted in AB formation, phosphorylation of tau protein and NFTs formation. They suggested that AMPK is a crucial pathway of captopril in autophagy regulation and a possible choice for investigation in Alzheimer’s disease [19, 66, 67].

**Fig. 2:**
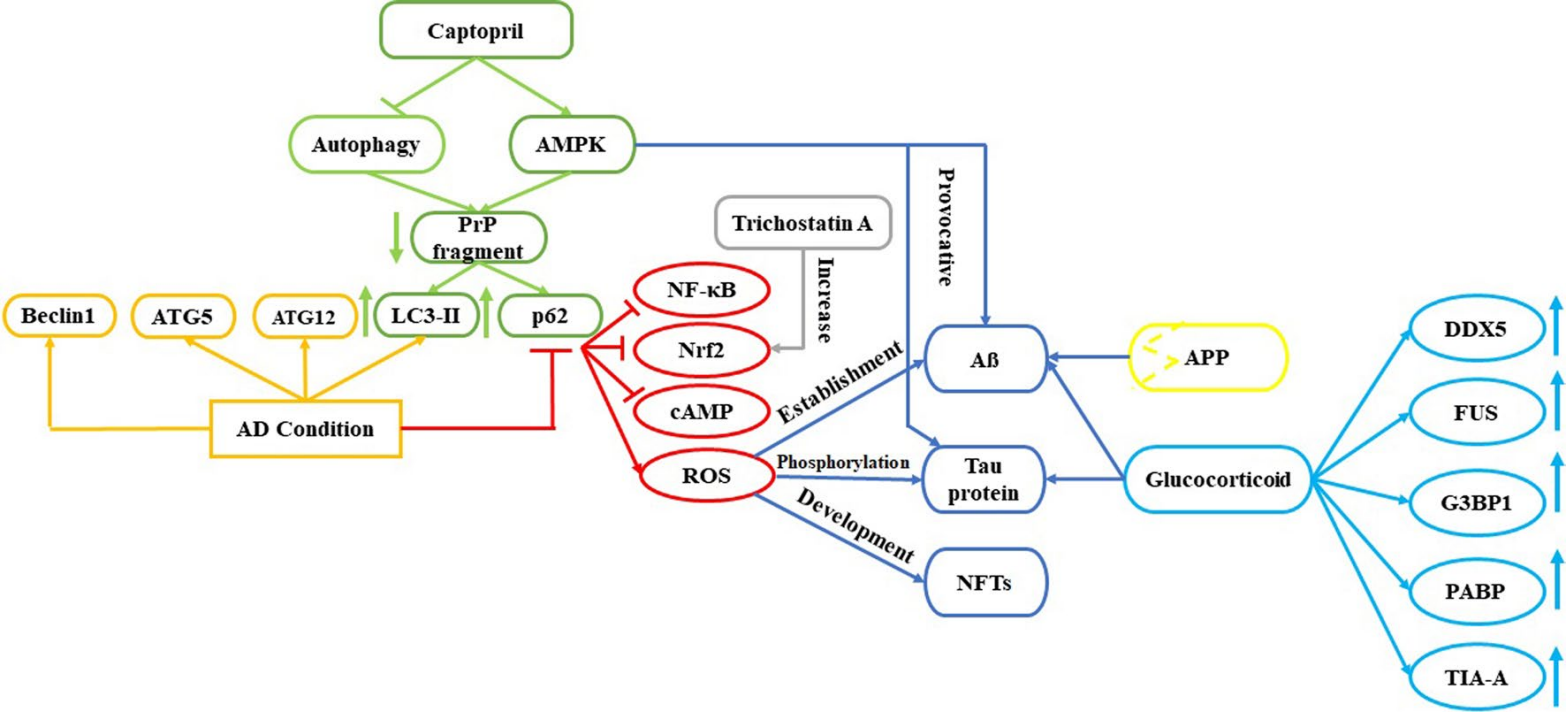
The interplay between autophagy and oxidative stress in Alzheimer’s disease. AD condiiton is acompained by increase in LC and inhibition of p62 expersions that inhibit the NFKB, Nrf2 and cAMP but increase ROS production

Weng and coworkers elucidated that the administration of camellia oil might alleviate AD pathogenesis through the microbiome-gut-brain axis and regulation of oxidative stress, autophagy, and inflammation [68] (Fig. 2.).

### Parkinson’s disease

Parkinson’s disease (PD) is a movement disorder with remaining clinical features including resting tremor, bradykinesia, rigidity, and postural instability. Based on some study some pieces of evidence display autophagy dysfunction and oxidative stress, implicated in the pathogenesis of PD, which leads to steadily loss of dopaminergic neurons in the Substantia Nigra. Autophagy is essential for the differentiation, growth, maintenance, and homeostasis of neuronal cells [69, 70].

Modulations of the expression of *BECN1* and *TFEB* as autophagy-related genes and regulation of numerous autophagy regulators, for instance, rapamycin, trehalose, lysosome modulators, etc., have exerted anti-PD implications in a variety of experimental models [71].

A number of genes are correlated to the primary pathology of PD, encompassing *α-synuclein*, *GBA*, *Parkin*, and *PINK1*. Autosomal recessive PD is connected to the taking place of mutations in *PINK1* and *Parkin*, which motivate damage in the degradation of injured mitochondria through activation of mitophagy [54]. Genetic erosion of *Pink1* led to a deficiency of striatal mitochondria respiration and weakness to oxidative damage in the neuronal cells [72]. Likewise, the ablation of *Parkin* resulted in dysfunctions that were attributable to synaptic plasticity and striatal mitochondria [73]. PD is also described by Lewy bodies in the neuronal nucleus, which contains protein aggregate of α-synuclein which are regarded as insoluble and degraded by CMA. Though, mutant *α-synuclein* has a great affinity with LAMP-2A, which hinders lysosomal uptake of the substrates, therefore stopping CMA-dependent degradation. Self-determining of the protein attachments, an amplified α-synuclein level damages autophagy, which results in *ATG9* mislocalization [74].

The α-synuclein is involved in the cytoplasmic clearance in autophagy responsible for Parkinsonism. The recent advancement in the study suggested that a generation of pathological abnormalities due to Beclin-1 overexpression causes low clearance of α-synuclein [75].

Mitochondria have a vital function in redox regulation of autophagy as the producer and scavenger of ROS. The dysfunction of mitochondria is a noticeable commencing factor of nervous system diseases which can be initiated by exceeding ROS. Mitophagy is considered a strategy for clearing the impaired mitochondria to restore homeostasis substantial pathological stress [25, 75].

Dagda et al*.* revealed that knockdown of *PINK1* in a recessive PD model could cause the accumulation of mitochondrial ROS in consort with clustered fragmented/depolarized mitochondria associated with autophagy [73]. Furthermore, it is noteworthy to discuss the crucial function of autophagy in the limitation of neuronal fate induced by RNAi knockdown of genes engaged in autophagy regarded PD [76].

Long noncoding RNAs (lncRNAs) have a prospective and versatile role in regulating autophagy engaged in PD by the mediation of diverse targets such as *PTEN1*, mTOR, etc. TFEB-mediated autophagy is a potent mechanism for survival throughout oxidative stress in PD. Zhuang and coworkers obtained valuable findings, including autophagy enhancing impacts of *Torin1* (an mTOR-dependent TFEB/autophagy promoter) and curcumin analog C1 (a TFEB-dependent and mTOR-independent autophagy promoter) in PD models [77].

Ning and coworkers demonstrated that β-asarone might regulate the ER stress-autophagy by impeding the PERK/CHOP/Bcl-2/Beclin-1 signaling pathway in 6-OHDA-induced parkinsonian rats [73, 78]. Numerous studies have indicated that mitochondrial dysfunction and mitochondrial complex Ι deficiencies also contribute to PD by aggregating oxidative damage in dopaminergic neurons, which can play a role in the initiation or progression of this disease. DJ-1, a contributing protein in the incidence of familial PD, is indispensable for moderating PINK/Parkin-mediated mitophagy. DJ-1 and DJ-1-binding complexes have been recognized for neuroprotection combat oxidative stress in PD rats [12, 79].

1-methyl-4-phenyl-1, 2, 3, 6-tetrahydropyridine (MPTP) by reduction of tyrosine hydroxylase (TH) protein and dopamine (DA) amount creates a PD model in an animal model. Furthermore, Ebert and colleague's study exhibited that Insulin-like growth factor-1 (IGF-1) had growth effects on deteriorating dopamine-producing neurons in a PD rat model and assist the durability of human neural progenitor cells (hNPC) in vitro and post transferring to rat PD model [80].

Exogenous IGF-1 as a neuroprotective agent has shown the potential to reduce neuronal lesions. In a study Wen-Fang and colleagues explored the IGF-1 effect on MPTP/MPP + induced neuronal damage. IGF-1 could down-regulate autophagy by hindering signaling of the PI3k-Akt-mTOR pathway and activation of G-protein coupled estrogen receptor (GPER). However, they indicated that more study is needed to understand this mechanism of action of IGF-1 in the CNS [81].

Apelin-36 as a neuroendocrine peptide combated MPTP-induced PD in mice that might be connected with mitigation of oxidative stress and the amelioration of LC3-II and Beclin1 and inhibition of p62 expression (82). Glucagon-like peptide-1, -2 protected neuronal cells in PD circumstance via improving *ATG7*, LC3B, and Beclin1 [83]. Poly (ADP-ribose) polymerase 1 (PARP1) signaling pathway, which is a regulator for oxidative stress, can regulate TFEB-mediated autophagy as a therapeutic approach to combat PD via amelioration of α-synuclein degradation [84].

Lactulose and melibiose upregulated levels of SOD2, Nrf2, and NQO1 regarding the oxidative stress process and improved the LC3-II/LC3-I ratio in the association of autophagy in the MPTP-ventral midbrain [85]. Guo et al*.* discovered that GM1 ganglioside has an anti-PD potential which is described as the ability to promote autophagy by the autophagosomes in the substantia nigra of PD mice along with alterations of LC3-II and p62 in the MPP + -treated SH-SY5Y cells, which are dependent on the removal of α-synuclein [86].

### Huntington’s disease

Huntington’s disease (HD) is a neurodegenerative disorder instigated by mutant proteins accompanying extended glutamine replications (polyQ) [87]. HD pathogenesis is intensely impacted by neuronal autophagy dysfunction. Huntingtin (HTT) is the most feasible polyQ protein for HD incidence. According to previous studies, HTT damages autophagosomes [76]. The wild type of HTT functions as a scaffold protein entailed in the signaling of several autophagy proteins to the autophagosome in the selective autophagic procedure. A loss of HTT results in the observation of failure in autophagosome transport and successively causes substrates degradation. Mutant (m) HTT also prevents a striatum-specific protein, Rhes, which interacts with Beclin1 to process autophagy. Tsunemi et al*.* claimed that peroxisome proliferator-activated receptor γ (PPARγ) coactivator 1α (PGC-1α), which is a regulator of oxidative stress and mitochondrial biogenesis, could improve HD hallmarks in a murine model. They discovered that PGC-1α promoted HTT throughput by activation of TFEB [88].

Prolonged polyQ regions adjust BECN1 deubiquitination and decline BECN1 protein levels. Hence they can inhibit starvation-induced autophagy. In addition, polymorphisms in *ATG7* are connected with the early onset of HD. Amelioration of autophagy via hindering mTOR facilitates the HTT aggregates’ clearance [89].

### Amyotrophic lateral sclerosis

Amyotrophic lateral sclerosis (ALS) is a NDD described by the occurrence of the selective fate of motor neurons. p62/SQSTM1 is a central factor responsible for the physio/pathological targeting of ALS [76]. It has been observed that C9ORF72 depletion via regulation of mTORC1, TANK-binding kinase 1 (TBK1), optineurin, valosin-contain protein, Cu/Zn superoxide dismutase (SOD1), ALS2 (Alsin), p62, vesicle-associated membrane protein-associated protein B (VAPB or ALS8), and sigma receptor-1 (SigR1 or ALS16) have participated in ALS and frontotemporal dementia (FTD) as autophagy-related NDDs [90, 91].

### Ataxia

Fragile X-associated tremor/ataxia syndrome (FXTAS) is a neurodegenerative condition accompanying a permutation repeat expansion (55–200 CGG repeats) in the 5′ noncoding region of the FMR1 gene. Ma et al*.* reported that the existence of elevated oxidative stress led to autophagy. Indeed, the study showed that exceedingly enhanced levels of conjugated small ubiquitin-related modifier 2 (SUMO 2) protein and p62/sequestosome-1 (p62/SQSTM1) protein were observed via autofluorescence-based fluorescence-activated cell sorting (FACS) and liquid chromatography/tandem mass spectrometry (LC–MS/MS)-based proteomics within the inclusions [92].

Spinocerebellar ataxia type 2 (SCA2) is a rare polyglutamine-dependent NDD affected by a CAG repeat expansion in the *ataxin-2* gene. Although in a recent study, it has been found that oligomerized *ataxin-2* and oxidative stress impact autophagic clearance in SCA2 cells; this happening is involved in the pathophysiology of SCA2, activation of autophagy or clearance of oligomers can verify to be beneficial approaches in treatment [93].

Autosomal recessive spastic ataxia of Charlevoix-Saguenay (ARSACS) is a sporadic early-onset neurological disease instigated by mutations in SACS, which encodes sacsin. Expression of LC3 decreased, and levels of p62 increased even after treatment with the lysosomal inhibitor bafilomycin A1, representing deficiency of the autophagic flux with correlation to oxidative stress and mitochondrial degradation [94]. Thus, the loss of sacsin is caused by oxidative stress and mitochondrial dysfunction, a novel mechanism in the pathogenesis of ARSACS [94].

### Spiral ganglion neuron degeneration

Dysfunction of autophagy is a significant factor in spiral ganglion neuron (SGN) degeneration. Thus, TFEB may be a possible objective for diminishing SGN degeneration subsequent sensory epithelial cell loss which can be oxidative stress in the cochlea of mice [95].

### Cerebral ischemia

L-glutamine (L-Gln), an amino acid, has a crucial intermediary role in mTORC1 activity and is a premier substance in some solute carriers (Slc), transmembrane transporters families like the Slc38a and SNAT1 families. L-Gln initiating mTORC1 signaling via the concurrent opposing flow of L-Gln and essential amino acids (EAAs) [96]. L-Gln are neuron-specific transporters and are an essential part of neurological disorders. However, their engagement in neurological disorders is still not wholly recognized and needs more studies. Sodium-coupled neutral amino acid transporter 1 (SNAT1) up-regulates mTORC1 activity, which induces neurological disorders like ischemic strokes to increase cell death. Ribosomal protein S6 kinase beta-1 (p70S6K1) regulates translation by phosphorylation of ribosomal protein S6 and p70S6K1itself phosphorylation by mTORC1, based on the phosphorylation level, can act as a successor for mTORC1. Comparing mRNA amounts of Slc transporters showed SNAT1 expression in the brain, particularly in neuronal nuclei (NeuN) positive neurons but not in glial cells. Inhibition of mTORC1, which initiated autophagy, improved cell protection and reduced neuronal damage in the ischemic mice model. So, because of the site-specific expression of SNAT1, this was proposed as a prospective target of subsequent therapies for neurological disorders [97–100].

Hamartin (Tsc1) augmented resistance to oxygen–glucose deprivation (OGD) and neuronal ischemia by inducing beneficial autophagy through a mTORC1-dependent mechanism [101].

Neuronal-targeted TFEB restored the impairments of the autophagy-lysosomal pathway and mitigated cerebral ischemic injury [102].

Studies on miR-497 show their intervention in the pathological process of CNS diseases, but the exact pathway is not entirely understood. Kunlin Jin and colleagues explored miR-497 agonist (agomir), the miR-497 antagonist (antagomir), and 3-Methyladenine (3-MA), as an autophagy inhibitor, in a rat model of cerebral ischemic attack. They reported that treatment with miR-497 antagonists decreased the infarction zone, altered ischemic stroke neuronal decay, and increased LC3 expression. Nevertheless, when 3-MA was used for treating ischemia in rats, opposing the miR-497 antagonist, inhibition of autophagy did not show neuroprotective effects and infarction zone reduction. The miR-497 antagonist could improve cerebral recovery by upregulating autophagy in an age-dependent order. Moreover, the efficacy of miR-497 antagonist in cerebral recovery and infarction zone reduction was less effective in older rats than in younger rats [76, 103].

Regarding the shreds of evidence reflected in published documents, venous thrombolysis and endovascular intervention are usual therapeutic strategies used for re-establishing the blood supply obligatory to improve nerve tasks in ischemic cerebrovascular disease. Nevertheless, together animal and clinical studies have publicized that reperfusion subsequent ischemia leads to surveillance of brain damage with more severity. The ischemia–reperfusion injury (IRI) may be characterized as a problematical pathologic condition encompassing numerous factors such as oxidative stress. Extensive damages attributed to mitochondria have been considered in IRI containing unbalanced swelling and crista fragmenting of mitochondria, in ischemic cerebrovascular disease, particularly throughout the acute phase. This impairment motivates mPTP to open unremittingly, leading to the occurrence of an alteration in ROS formation, energy shortage, and membrane potential, thus prompting autophagy. It has been witnessed that autophagy is caused in the mouse striatum and cortex subsequent cerebral hypoxic-ischemia and augmented by a consequent ROS overproduction. Oxidative damage in mouse striatum and cortex subsequent cerebral hypoxia–ischemia causes autophagy. Autophagy during this circumstance can noticeably save neurons in the ischemic condition by inhibiting necrosis and apoptosis via abolishing damaged mitochondria [104].

It has been conveyed that insufficiency of fatty acids (FAs) intensely alters ischemia-induced autophagy activation. This is revealed by the raised levels of LC3 and Beclin1 expression, along with significant growth in 8-OHdG, signifying that FAs deficiency can ameliorate the levels of autophagy via induction of oxidative damage. A study has presented that together ROS and autophagy are involved in reperfusion injury afterward cerebral ischemia which antioxidants frequently stimulate autophagy [104]. The use of antioxidants well-identified as revulsive of autophagy has the ability to attenuate neuronal damage and expressively alleviate the infarcted area. Therefore, we will wonder that antioxidants might play a defensive character in ischemic injury by prompting autophagy. There could also be some more complex crosstalk mechanisms concerning autophagy and oxidative stress in the necessity of additional research [105].

Furthermore, SIRT3 may be a well-maintained deacetylase connected to biological roles like stress resistance, mitochondrial redox homeostasis, and energy metabolism. Furthermore, it can positively regulate autophagy over the AMPK-mTOR pathway, which endorses neuronal persistence in an in vitro oxygen and glucose deprivation (OGD) model of cerebral ischemia made by decreasing H_2_O_2_ and O_2_^−^. Pharmacological or genetic impeding of autophagy can amend SIRT6-mediated neuronal damage, feasibly via mitigating Akt signaling allied with oxidative damage in the OGD model of SH-SY5Y neurons. Besides, moderate activation of ROS can encourage Parkin translocation into the injured mitochondria, afterward suffer Parkin-mediated mitophagy and certify the mitochondria integrity in ischemic brain injury [106].

### Chronic cerebral hypoperfusion

Reduced blood flow is linked to many CNS diseases, like Alzheimer’s disease, atherosclerosis, and cerebral small vessel disease. So, as a blood flow reduction situation, chronic cerebral hypoperfusion is a problem for treating many debilitating cerebral disorders, leading to apoptotic neuronal cell death and cognitive disorders. Hence, for decreasing apoptotic neuronal cell death, a treatment for chronic cerebral hypoperfusion could be a good idea [76].

Su and colleagues explored cannabinoid receptor agonist WIN55,212–2 (WIN) and fatty acid amide hydrolase inhibitor URB597 (URB) in a rat model to be considered for treating chronic cerebral hypoperfusion induced apoptosis. They found that WIN and URB treated rats showing better action in memory tests and neural cells counting, proteomics, genomics, and brain sections analysis showed a reduction of neurotoxicity, phosphorylation of c-Jun N-terminal kinases (JNK), caspase-3 initiation, and Bcl-2 to Bax ratio [107–109]. In another study by this team, they investigated the URB effect on up-regulated autophagy in chronic cerebral hypoperfusion status in the brains of a rat model of chronic cerebral hypoperfusion; they reported that inhibition of autophagy by URB reduced autophagosomes accumulation and decreased synaptic degradation caused by excessive autophagy, and diminished mitochondrial dysfunction and mitophagy. Based on increasing phosphorylated *Akt* and mTOR levels and unchanged AMPK, they suggested Akt-mTOR signaling as a possible pathway to inhibit autophagy by URB [110].

### Spinal cord injury

A meta-analysis about the effect of autophagy regulation on spinal cord injury improvement showed it altered neurological recovery, whether it is increased or decreased, but no apparent discrepancy was seen among increment and decrement of autophagy in spinal damage treatment in a rat model [111].

Zhu et al*.* discussed that acupuncture could increase mTORC1expression in the peri-infarct cortex and decrease *ULK1*, *ATG13*, and Beclin1 amounts. However, above and beyond, acupuncture attenuated *LC3-II* and Beclin 1 expression and the number of autophagosomes [112].

Transcription factor E3 (*TFE3*) has excellent potential for use in ROS-mediated autophagy dysfunction following spinal cord injury. *TFE3* has been regulated partially afterward the spinal cord injury via regulation of AMPK-mTOR and AMPK-SKP2-CARM1 signaling pathways [61].

## Therapeutic implications of autophagy dysregulation

**Fig. 3:**
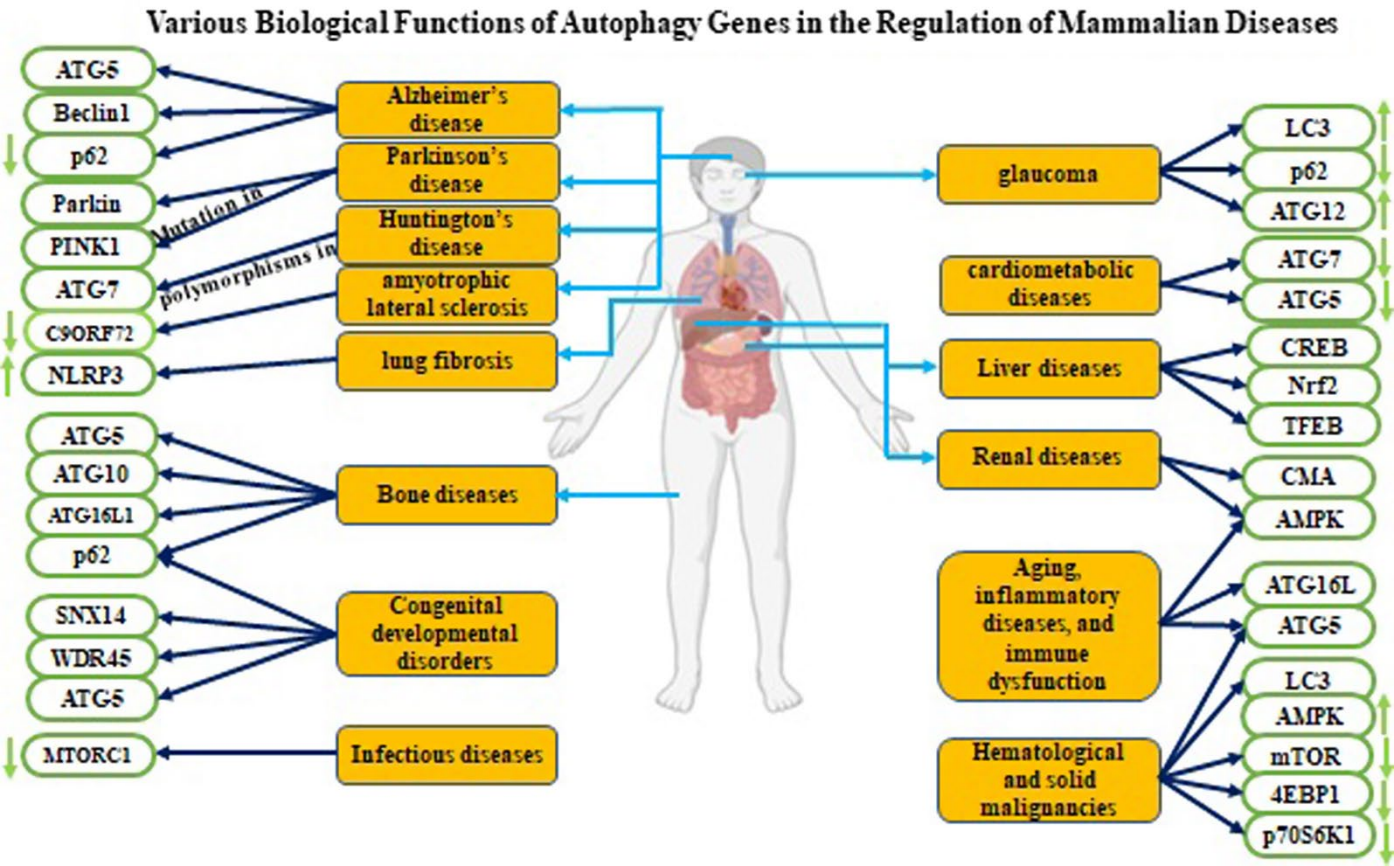
A variety of autophagy implications are exhibited in this figure. Activation/inhibition of autophagy and its related genes can contribute to neurological disorders, various cancers, cardiometabolic diseases, pulmonary conditions, aging, autoimmune disease, bone, skeletal muscle diseases, eye disease, congenital developmental disorders, and infections, inflammatory maladies, liver, and renal disease

The induction of therapeutic autophagy acts as a survival mechanism. However, progressive autophagy, also known as autophagic cell death, can lead to non-apoptotic cell death. Many human diseases such as autoimmune diseases, cancers, cardiovascular disorders, microbial infections, metabolic diseases, inflammatory responses, bone diseases, renal ailments, liver disorders, NDDs, etc., have been studied. Autophagy can be utilized both in maladaptive and adaptive functions in the pathogenesis of various diseases (Fig. 3.) [113].

### Microbial and viral infections, and parasites

Khandia and coworkers explained the roles of autophagy in infectious diseases in their recent review article very carefully. Autophagy has a critical role in viral infections, for instance, bird flu, swine fever, Ebola virus disease, Zika virus infection, SARS, Chikungunya infection, Dengue virus infection, viral encephalitis, Crimean-Congo hemorrhagic fever (CCHF) virus, Hendra virus infection, Nipah virus infection, and the West Nile virus infection [114]. Autophagosome formation is induced in the early stages of the influenza A virus. However, later stages result in the inhibition of autophagosomal maturation. MTORC1 downregulates classical swine fever virus replication through autophagy and IRES-dependent translation. Pathogenicity of the Dengue/Zika virus is controlled by antibody-dependent enhancement, which can cause autophagy induction in human umbilical vein endothelial cells. Transcription of *ATGs* is promoted in severe acute respiratory syndrome (SARS)-coronavirus. Moreover, it has been reported that endoplasmic reticulum (ER) stress in Dengue virus (DENV) infections led to autophagy activation, viral replication, and pathogenesis. Autophagy has also been shown to have a crucial role in microbial infections, including those caused by *Listeria*, *Salmonella*, *Shigella*, *Streptococcus*, *Mycobacterium tuberculosis*, and *Salmonella enterica* serovar Typhimurium [115]. The role of autophagy in Toxoplasmacidal mechanisms has been observed [116].

### Hematological and solid malignancies

Autophagy is more involved in cancers than other diseases; the effects of autophagy on cancer are highly dependent on the situation. It can eliminate the tumor or be ineffective or even strengthen the spread of the tumor. In recent years many studies have been conducted to investigate the use of autophagy to treat diseases. However, the critical question of whether autophagy could be regulated for cancer treatment or not remained unanswered, although there are now pieces of evidence that autophagy in cancer acts as a lifeline to chemotherapy. Although, studies have shown that autophagy is a vital cell rescue mechanism for clearing defective proteins and damaged organelles that conserve cell energy and function [21, 117, 118].

Plumbagin, a quinonoid component isolated from *Plumbago zeylanica* L., saikosaponin-d (Ssd) a triterpenoid saponin, berberine, carnosol, bufalin, licarin A isolated from *Myristica fragrans*, and isoliquiritigenin a flavonoid of *Glycyrrhiza uralensis* are examples of valuable phytochemicals which can induce cytotoxic autophagy in cancer cells [119].

The roles of miRNAs in osteosarcoma in recent investigations showed miRNAs like *MiR-22* involved in determinant sectors of autophagy gene regulation. *Beclin1*, *LC3*, Metadherin (*MTDH*), *ATG5* genes, and the proteins of *LC3*, *ATG5* that are closely related to autophagy regulation and anticancer drug resistance, when an encounter with miR-22 downregulate obviously [120–122].

Lovastatin inhibition of autophagy in glioblastoma cells which were resistant to temozolomide, enhanced efficacy of temozolomide through cessation of autophagy cascade by inhibition of *LAMP2* and dynein proteins. Impermanent formation of autolysosomes increased glioblastoma cell death [123, 124].

Proof of bufalin-induced autophagy comprised the formation of the acidic vesicular organelles, augmentation of autophagolysosomes, and accumulation of LC3-II. More reports presented that the mechanism of bufalin-induced autophagy connected with ATP depletion involved an increased AMPK activation, diminished phosphorylation of mTOR, and its downstream targets *4EBP1* and *p70S6K1* [125].

Autophagy inhibition in lung cancer can overcome drug resistance, like the effect of candesartan and gingerol on TRAIL-resistant lung cancers via autophagy inhibition caused a reduction of drug resistance [126, 127].

A study on the effect of cigarette smoke extract (CSE) on fibroblast cells of the lung seen CSE induction of autophagy and related proteins, such as optineurin. Fibroblasts in the following showed a rise in the amount of IL‐8, which caused elevation of cell malignancy potential. Inhibition optineurin reversed the pathway and reduced p62 protein and IL-8 expression in these treated fibroblasts. Thus, it seems that the IL‐8 expression of cigarette smoke triggered autophagy can support the spread of cancerous lung cells [128].

Zhang and colleagues explored the linkage of autophagy to the effectiveness of chemotherapy with a regimen of 5-FU and 3-MA in the treatment of Squamous cell carcinoma (SCC). SCC is a type of malignant skin cancer, and its prevalence is one-fifth of non-melanoma skin cancers. Thus, the treatment and prevention of SCC recurrence are among dermatologists' and radiotherapists’ fields [129].

3-Methyladenine (3-MA) is an inhibitor of autophagy by preventing the formation of autophagosomes and increases the sensitivity of SCC to radiotherapy, and increase the antitumor effect of 5-Fluorouracil (5-FU); in another study by Zhang and colleagues, they combined 5-aminolevulinic acid-photodynamic therapy (ALA-PDT) with 5-FU and found that this treatment regimen with or without 5-FU or 3-MA Stimulates apoptosis, primarily if used with 5-FU and 3-MA, this treatment regimen on cell lines of A431 and A375, the repressed proliferation of cells and ALA-PDT induced apoptosis and this effect increased by 3-MA pretreatment, which seems autophagy regulation is involved in ALA-PDT action [130].

Yi Zhang and colleagues explored the effect of sodium selenite on leukemia. They found p53 was a primary up regulator of phospholipid scramblase 1 (PLSCR1), acting as a confluence shifting autophagy to apoptosis in leukemia. PLSCR1 Tuning of apoptosis and autophagy possibly is the primary mechanism in the efficacy of chemotherapy [76, 131]. Ectopic expression of Beclin1 in breast cancer cells (MCF-7) activated autophagy. Moreover, the monoallelic deletion of Beclin1 was witnessed in numerous specimens of human breast, ovarian, and prostate cancer cells [132].

UVRAG, an upregulating agent of the Beclin1-class III PI3K complex, suppressed the proliferation and tumorigenicity of colon cancer cells. In addition, knockout of Bif-1which interacted with Beclin1 and activated the Beclin1-class III PI3K complex, ameliorating spontaneous tumors' progression in vivo [132–134]. Zhao and coworkers indicated that miR-375/sorafenib in lipid-coated calcium carbonate nanoparticles might play a significant role in the therapeutic approach of hepatocellular cancer via inhibition of autophagy [63]. A systemic mosaic deletion of *ATG5* or a liver-specific *ATG7* deficiency in mice led to the development of benign liver tumors, proposing the critical role of autophagy in the abrogation of spontaneous tumor genesis [135].

### Aging, inflammatory diseases, and immune dysfunction

Autophagy decreases during the aging process. It seems that maintaining autophagy is an effective anti-aging treatment. During the aging process, the innate immune system response to external antigen decreases, ROS production and IL-1B concentration, and IL-18 Increase by macrophages. Other innate immune cells that are affected by aging are neutrophils. Although there is no reduction in the total number of these cells during aging, studies have shown that their capacity for phagocytosis and xenophagy have an inverse relationship with age. They find similar conditions diminish antigens presentation and chemotaxis. In addition to the weakening of immunity against pathogens, chronic inflammation develops, and immune cells do not return to the basal level of homeostasis after inflammation. Peripheral cells such as adipose increase inflammatory status by increasing the release of cytokines. Cell-mediated immunity because of DNA damages and shortening of telomeres have obstacles to replicating and reproducing. Their ability to create a memory is reduced [136, 137].

T cells secrete proinflammatory cytokines without antigen stimulation, do not respond to apoptotic signals, diminished the production of the central cytokines like TNF-α, IL-2, INF-γ, and granzyme B, and consequently, their response to infections decreases. Although B lymphocytes are less studied in aging research, in some studies on human vaccination, due to reduced humoral immune response, it appears to be related to B cells malfunction [76, 138]. Furthermore, macrophages, B, and T cells for phagocytosis, antigen presentation, and hemostasis require autophagy. Therefore, it is conceivable to reduce the immunological effects of aging by targeting autophagy [139].

Recent studies indicated that AMPK activation inhibited cell-induced aging by boosting activation of autophagy, detention of mTORC1 process, and reducing oxidative stress. Hence, it seems to be an excellent strategy to enable AMPK to stop cell aging [140, 141].

*ATG16L* and autophagy stimulatory immunity-related GTPase (*IRGM*) are two genes involved in autophagy regulation related to Crohn’s disease. *ATG2B* and autophagy have been regulated by miR-143 in another study of Crohn’s disease [142]. Blocking the differentiation and function of Treg cells by the interleukin-21/mTOR axis via autophagy inhibition in systemic lupus erythematosus patients has been targeted. Moreover, the *ATG5* gene has participated in the incidence and development of systemic lupus erythematosus as an autoimmune disease [143]. Additionally, autophagy and chronic inflammation involved cystic fibrosis, an incurable genetic condition initiated by mutations in the gene encoding the cystic fibrosis transmembrane conductance regulator. Moreover, the modulation of autophagy is essential in other inflammatory diseases like Behcet’s disease and celiac disease [144, 145].

### Pulmonary diseases

Meng and colleagues claimed autophagy could mitigate pulmonary fibrosis by mediating the activation of the NOD-like receptor family pyrin domain containing 3 (NLRP3) inflammasome induced by angiotensin II-mediated ROS via modulation of redox balance [146]. Moreover, asthma and COPD can be related to the role of autophagy [144].

Evidence indicated that autophagy plays a central role in tissue remodeling. The changed autophagy pathway in response to cellular stress in asthma and COPD resulted in activation and crosstalk between structural airway and immune cells. This further leads to autophagy impairment causing intracellular constituents degradation and secreting inflammation mediators, which cause lung airway remodeling [144].

### Ocular diseases

Corneal oxidative stress upregulated *LC3*, *Beclin 1*, and *ATG12* levels; however, *P62* were downregulated. Hence, various signaling pathways, such as the mTOR, showed the interplay of ROS and autophagy in corneas [147]. Regulation of the expression of *LC3II* and *P62* regarding suppression of the senescence of lens epithelial cells by restoring autophagy flux after metformin administration was observed. More studies about the role of autophagy in glaucoma have been reported [144]. Autophagy can act as a cyto-protector or a cyto-killer in glaucoma. The dual role of autophagy in glaucoma progression is related to the glaucoma stage, autophagy stage, autophagy detection time, and even genomic changes [144].

### Cardiometabolic diseases

Cardiometabolic disease comprises a wide range of cardiovascular diseases, diabetes, and obesity [148]. In vitro and in vivo results elucidated that hindering the expression of *ATG7* had a defective role regarding insulin signaling and augmented ER stress. On the contrary, reinstatement of *ATG7* expression in the liver limited ER stress and improved insulin activity and systemic glucose tolerance in mice with obesity. Furthermore, autophagy has a crucial role in normal adipogenesis, and abridge of autophagy has anti-obesity and insulin-sensitizing properties [149, 150].

According to the shreds of evidence, impaired autophagy might contribute to insulin deficiency and hyperglycemia. Autophagy was considered a significant regulator in pancreatic β-cells, and *ATG7* mutation exhibited damaged glucose tolerance. In an investigation on the effect of liraglutide on cardiac fibrosis caused by aortic banding in rats, results indicated that inhibition of *p70S6K*, a ribosomal protein S6 kinase, and mTOR signaling by liraglutide, showing a positive effect on reduction of heart dysfunction emanating from fibrosis and cardiomyocyte hypertrophy by increase autophagy [151]. *ATG5* deficiency was reported to contribute to various cardiovascular diseases [126].

### Renal diseases

Dysregulation of autophagy is one of the reasons responsible for the pathogenesis of acute kidney injury, or incomplete kidney repair after acute kidney injury and chronic kidney disease of diverse aetiologies, comprising diabetic kidney disease, focal segmental glomerulosclerosis, and polycystic kidney disease. Autophagy also plays a critical role in kidney aging. CMA, AMPK, sirtuins, and the mTOR pathway are considered the most feasible gates for regulating autophagy-related to renal disease [11, 144, 152].

### Liver diseases

Autophagy has an emerging primary function in maintaining the balance of liver metabolism and the modulation of its physiology. On the contrary, numerous documents have shown that autophagy may disease-dependently play a part in the pathogenesis of liver disorders, like Wilson's disease, α-1 antitrypsin deficiency, hepatocellular carcinoma, cirrhosis, acute liver injury, chronic alcohol-associated liver disease, liver hepatitis, fibrosis, steatosis, and non-alcoholic fatty liver disease (NAFLD) [153]. Carbamazepine, rapamycin, and ursodeoxycholic acid are drugs with autophagy induction and assistive in combating various liver diseases [153]. There are a number of transcription factors that can assist in regulating hepatic functions related to autophagy. These transcription factors are encompassing CREB, Nrf2, PPARα, and TFEB. Ueno et al*.* demonstrated that the mTORC1 complex acts as a fundamental portion of the liver's amendment to metabolic disorders, containing nutrient starvation. Inactivation of mTORC1 causes TFEB activation and NCOR1 inactivation. Moreover, the integral functioning of Nrf2 and Parkin-mediated mitophagy have participated in the modulation of autophagy in liver disorders [144, 154–156].

### Bone diseases

Autophagic catabolism is involved in controlling and managing the survival and working of osteoclasts, osteocytes, and osteoblasts. Hence it is essential for the conservation of skeletal homeostasis. Unusual autophagic action causes obviation of the balance of the bone-remodeling manifesting as pathological conditions, comprising osteopetrosis and osteoporosis. Regulation of autophagy has eliminated therapeutic potential in the treatment and prevention of bone-related diseases. *ATG5, ATG10*, *ATG16L1*, and *p62/SQSTM1* are significant targets that can be attributed to Paget's disease [144, 157, 158].

### Skeletal muscle disorders

According to the pivotal character of skeletal muscles in metabolism control, maintaining the muscle homeostasis through making balance among catabolic and anabolic processes is of high emphasis. Hence, autophagy is a principle catabolic mechanism in the skeletal muscles which helps in the achievement of the mentioned aim. The significance of autophagy as a helpful target and recommend explaining associations between protein unfurling and mTOR-dependent or mTOR-independent hypertrophic reactions is likely to uncover particular helpful windows for treating muscle squandering clutters (muscle function loss or paralysis) [19, 144].

### Congenital developmental disease

While the appropriate function of autophagy is vital for the exact function and CNS development, any single gene disorder related to autophagy signaling can be harmful. Some of the gene disorders of autophagy signaling are stated as *EPG5*-associated Vici syndrome, *SQSTM1/p62*-associated childhood-onset neurodegeneration, *SNX14*-associated autosomal-recessive spinocerebellar ataxia 20, *WDR45*-related β-propeller protein-associated neurodegeneration, *ATG5*-associated autosomal-recessive ataxia syndrome, and numerous types of hereditary spastic paraplegias [144, 159, 160].

## Conclusion

In summary, regarding the emerging emphasis on cellular autophagy, a comprehensive literature overview was conducted to discuss the pharmacological aspects of autophagy, focusing on its interplay with oxidative stress in neurological disorders. Various human diseases have been contributed to alterations in autophagy entailing cancers, cardiometabolic diseases, renal and liver diseases, bone diseases, neurological disease, aging, and immune dysfunctions. This study claimed some of the principal interrelations of autophagy and oxidative stress in cerebral ischemia, Huntington’s disease, Parkinson’s disease, Alzheimer’s disease, cerebral hypoperfusion, and spinal cord injury. This interplay assists in the foundation of variable targets for drug discovery.

## Data Availability

The datasets used and/or analyzed during the current study are available in the body of main text.
